# Clinical Efficacy of TM02^®^ in Allergic Rhinitis: Significant Symptom Alleviation Associated with IL-5 Downregulation and Reduced Allergic Inflammation

**DOI:** 10.3390/cimb48090898

**Published:** 2026-09-02

**Authors:** Hao-Dong Tan, Sien-Hui Tan, Boon-Hong Kong, Chee-Kuan Wong, Revadi Govindaraju, Tengku Ahmad Shahrizal Tengku Omar, Shin-Yee Fung

**Affiliations:** 1 Medicinal Mushroom Research Group (MMRG), Department of Molecular Medicine, Faculty of Medicine, Universiti Malaya, Kuala Lumpur 50603, Malaysia; haodong@um.edu.my; 2 Department of Otorhinolaryngology, Faculty of Medicine, Universiti Malaya, Kuala Lumpur 50603, Malaysia; shtan@ummc.edu.my (S.-H.T.); tshahrizal@ummc.edu.my (T.A.S.T.O.); 3 Division of Respiratory Medicine, Department of Medicine, Faculty of Medicine, Universiti Malaya, Kuala Lumpur 50603, Malaysia; ckwong@um.edu.my; 4 Subang Jaya Medical Centre, Subang Jaya 47500, Malaysia; grevadi@gmail.com

**Keywords:** allergic rhinitis, randomized controlled trial, double-blind, placebo-controlled, *Lignosus rhinocerus* TM02^®^, clinical efficacy, safety

## Abstract

Allergic rhinitis (AR) is a chronic inflammatory disease that substantially affects quality of life. Despite available therapies, symptom control remains suboptimal owing to treatment burden. Cultivated sclerotium of *Lignosus rhinocerus* (TM02^®^), known for its allergy-dampening bioactivities, is a promising adjunctive AR treatment. A randomized, double-blinded clinical trial was conducted in 108 AR patients, who received either TM02^®^ or placebo for 30 days. Primary outcomes consisted of Total Nasal Symptom Score (TNSS), reflective of past 12 h and 2 weeks (TNSS12hrs and TNSS2wks), and the Allergic Rhinitis Control Test (ARCT). The secondary endpoints were the peripheral immune biomarkers: IgE, IFNγ, IL-4, IL-5 and IL-13. Safety was assessed with adverse events (AEs) and full blood-counting analyses. Statistical analysis was performed using generalized estimating equation (GEE) in the modified per-protocol (mPP). mPP analysis (N_TM02^®^ = 25; N_placebo = 31) demonstrated significant treatment effectiveness of TM02^®^ in total TNSS12hrs, total TNSS2wks, and ARCT (β = −3.70, *p*-value < 0.001; β = −4.44, *p*-value < 0.001; and β = 3.89, *p*-value = 0.017, respectively). Secondary endpoint analysis did not reveal significant changes. However, a positive associative pattern (β = 14.94; *p*-value = 0.003) was observed between TNSS2wks and IL-5 level changes. There were no significant differences in AE incidences and laboratory safety biomarkers between treatment arms. The present study revealed that TM02^®^ reduced AR symptom severity with minimal adverse effects. The underlying mechanism may involve the modulation of IL-5 levels and allergic inflammation. Overall, TM02^®^ is a potential adjunctive or complementary treatment option for AR with persistent symptoms.

## 1. Introduction

Allergic rhinitis (AR) is a pervasive, chronic inflammatory disease that significantly encumbers health and the economy [1,2]. With a prevalence of approximately 15% to 25% worldwide, this upper respiratory tract disease is characterized by nasal symptoms such as nasal congestion, sneezing, rhinorrhoea, and nasal itchiness. The condition is driven by inappropriate immunologic responses of the upper nasal cavity towards common environmental allergens [2,3]. Within the Asia–Pacific in 2018, it has been estimated that the expenditure on AR treatment had reached as high as 105.4 billion USD [1].

The molecular pathogenesis of AR is characterized by two distinctive stages of allergic responses, associated with different clinical manifestations. Upon exposure to sensitized allergens, the early-phase response, characterized by IgE-mediated mast cell and basophil activation, results in the release of pro-inflammatory mediators such as histamine, leukotrienes, and prostaglandins, ultimately leading to increased vascular permeability, hypersecretion, and sensory nerve stimulation [4]. Clinically, this results in the initial onsets of pruritus, sneezing, and rhinorrhoea. After four to six hours, the immunologic profile transitions to that of the late-phase response, characterized by granulocytes and T helper 2 (Th2) cells’ infiltration and activation, which are associated with sustained allergic inflammation [5,6]. During this process, the release of eosinophil granules into the inflamed tissue enables the promotion and enhancement of other immune cells, in an IgE-independent manner [5]. Ultimately, this leads to tissue-level damages, characterized by tissue scarring.

The current treatment landscape for AR comprises oral antihistamines (AH), intranasal corticosteroids (INCS), and, in selected patients, allergen immunotherapy (AIT) [6]. These treatment options are capable of blunting the early and/or late immune responses, preventing the cascade and perpetuity of allergic inflammation. While these treatment alternatives provide the effective control of nasal symptoms, their long-term acceptability may be limited by undesirable adverse effects, such as somnolence, attention disturbance or nervousness in the case of AH, epistaxis, and nasal septum perforation in the case of INCS, and urticaria, dyspnoea, or dizziness in the case of AIT [7,8,9,10].

Tiger Milk mushroom, the cultivated *Lignosus rhinocerus* strain (TM02^®^), is a promising adjunctive or complementary treatment option for AR. *L. rhinocerus* belongs to the Polyporaceae family, and is a popular medicinal mushroom with a long history of usage by natives in Southeast Asia and, especially, by the indigenous communities of Peninsular Malaysia [11]. Traditionally, these indigenous communities, apart from the Malays and Chinese in Malaysia, have utilized concoctions of this mushroom to treat a variety of diseases, including cough, asthma, fever, chronic hepatitis, gastric ulcer, liver, and breast cancer, and as a general tonic [12]. In a more recent market survey, pronounced allergy-alleviating effects have been posited by consumers, where nasal allergy symptom reductions and breathing improvements have been reported [11].

From 2002, the Medicinal Mushroom Research Group (MMRG) (Faculty of Medicine, Universiti Malaya) has conducted numerous works using TM02^®^, unveiling scientifically robust data that supports the use of TM02^®^ research in modern practical usage. Such includes extensive safety research, where TM02^®^ has been proven safe for consumption [13,14]. Additionally, TM02^®^ has also been subjected to corticosteroid screening by the Toxicology Laboratory, National Poison Centre, with results confirming the absence of corticosteroids (dexamethasone, betamethasone, cortisone, hydrocortisone, prednisone, prednisolone, methyl prednisolone, triamcinolone) in the mushroom. Beyond safety research, bioactivities of the mushroom have been extensively studied in *in vitro* and *in vivo* contexts, and being of particular interest, the extract from TM02^®^ (xLr^®^) and its fractionhave been demonstrated to exhibit immunomodulatory and anti-inflammatory activities [15,16,17]. Furthermore, a prior clinical trial on asthma has been conducted, demonstrating significant reduction in blood eosinophil count and an absence of significant adverse effects [18]. Given empirical evidence highlighting its efficacy and safety in treating atopic diseases, TM02^®^ was selected for testing in the clinical setting for the treatment of AR. We hypothesize that TM02^®^ may alleviate symptoms in patients with allergic rhinitis by modulating allergic inflammation. Specifically, we hypothesize that treatment with TM02^®^ will result in an improvement in allergic rhinitis symptoms and modulation of cytokine levels. The study will also evaluate the safety and tolerability of TM02^®^ in patients with allergic rhinitis.

## 2. Materials and Methods

### 2.1. Study Design and Participants

This randomized, double-blinded, placebo-controlled clinical trial was conducted at Universiti Malaya Medical Centre (UMMC) in Kuala Lumpur, Malaysia, from September 2024 until December 2025, with a 30-day follow-up period. The Medical Research Ethics Council of UMMC approved the study protocol (MREC ID NO: 2024222-13450) on 13 June 2024. The study was retrospectively registered in the International Traditional Medicine ClinicalTrial Registry (ITMCTR) ITMCTR2026001939 on 2 August 2026.

All the participants provided written informed consent for their participation in the study. This study was conducted in accordance with the principles of the Declaration of Helsinki and complied with Malaysian Good Clinical Practice guidelines [19,20].

The main inclusion criteria were as follows: (1) patients aged above 18 and below 60 years; (2) non-smoker; (3) mild-to-moderate/severe persistent AR that is being treated with a standard treatment of intranasal corticosteroid and antihistamines with or without antileukotrienes; (4) participants that can fully understand the aim of the study and its protocol and are able to give consent.

The main exclusion criteria were as follows: (1) patient that is on other health supplements.; (2) patient that is on macrolide therapy, immunotherapy, or biologics for upper or lower airway diseases; (3) pregnant and lactating women; (4) chronic rhinosinusitis with or without nasal polyps; (5) sinonasal tumors; (6) previous sinonasal or skull base surgery; (7) other autoimmune disease that is on immune-modulating drugs; (8) presence of severe co-morbid medical illness such as end stage renal failure, heart failure, poorly controlled diabetes mellitus and uncontrolled hypertension; (9) history of drug allergy or anaphylaxis.

### 2.2. Sample Size Calculation

Sample size was calculated using G*Power 3.1.97. Using a Type 1 error (α) of 0.05 and power of 90%, a sample size of 45 participants for each arm was needed to detect an effect size of 0.15. Assuming an attrition rate of approximately 15%, a total sample of 108 subjects was required for this study.

### 2.3. Investigational Medicinal Product and Randomization

The investigational medicinal product (IMP) consisted of TM02^®^ capsules and identical placebo capsules. Each TM02^®^ capsule contained 300 mg freeze-dried sclerotial powder of *L. rhinocerus* TM02^®^ (Batch no.: E001C22J), cultivated and processed by LiGNO Biotech Sdn. Bhd. (Selangor, Malaysia). The TM02^®^ strain has been previously verified using PCR amplification of its internal transcribed spacer (ITS) region, and a sample has been deposited at the Royal Botanic Gardens, Kew (London, UK) with specimen voucher number: (K(M) 177812) [21]. Additionally, it is registered under the New Plant Variety Act (NPVA) by the Ministry of Agriculture, Malaysia (Registration No. PBR0182) and under the National Pharmaceutical Regulatory Agency (NPRA) (MAL20116109TC).

Preclinical studies have suggested a dosage of 500–1000 mg per day to be sufficient for bioactivity manifestation, while safety studies conducted at doses up to 2000 mg/kg body weight have demonstrated that TM02^®^ has a high margin for safety consumption [13,14,22]. Each capsule is 300 mg, and the dosage of 1200 mg has been determined to be the optimal median dose based on feedback from a post-marketing survey of TM02^®^ consumers.

The patients were randomized to the placebo arm or the TM02^®^ arm. Patients were required to take four capsules (4 × 300 mg) daily in two separate intakes, post-prandial, for a duration of 30 days. The placebo consisted of identically sized capsules containing powdered brown rice that were indistinguishable in color from the TM02^®^ capsules. Block randomization with a block size of four was utilized, and participants were sequenced according to these blocks. This study utilized a double-blind design ensuring both participants and investigators involved in the study were blinded to treatment assignment.

### 2.4. Study Endpoints

The primary endpoints were the patient reported outcomes, which were the Total Nasal Symptom Score (TNSS) and Allergic Rhinitis Control Test (ARCT). The TNSS employed, is a validated reflective questionnaire, measuring severity of nasal symptoms [23,24]. Five common nasal symptoms were included, which were nasal congestion, nasal itchiness, sneezing, runny nose and sleep disturbances. For each of these symptoms, patients were to assign a four-scaled response, ranging from 0 (none), 1 (mild), 2 (moderate) to 3 (severe), in the past 12 h (TNSS12hrs) and in the past two weeks (TNSS2wks). A composite score, from the summation of the total symptom severity in the past 12 h (TNSS12hrs total) and in the past 2 weeks (TNSS2wks total) were then constructed and subsequently analyzed. The ARCT is a validated questionnaire that assesses the level of control that patients have towards allergic rhinitis [25]. It comprises five items, each scoring from 1 to 5, with 5 indicating the highest levels of control. The sum of these scores constitutes the ARCT total score and was subsequently analyzed. Both the TNSS and ARCT questionnaires were administered to the patients at visit 1 (baseline) and visit 2 (after 30 days). The measurement items were distributed to the patients at the start of each visit, with their responses collected by the end of each visit. Treatment effectiveness was evaluated by investigating the changes in primary endpoint measures between visit 1 and visit 2, and its association with treatment allocation.

The secondary endpoints of the present study include serum IgE and plasma cytokine investigations. Peripheral blood samples were collected during visit 1 and visit 2, using silicone coated serum and EDTA Vacutainer^®^ tubes (Becton Dickinson UK Ltd., Plymouth, UK) for each visit. The serum samples were sent to the UMMC clinical diagnostics labs for serum IgE quantification within 2 h of blood collection while blood samples in EDTA tubes were spun at 1500× *g* for 15 min at 20 °C to isolate plasma, which were stored in −80 °C. These selected plasma samples were subjected to automated immunoassay platform, ELLA simple plex, for IFNγ, IL-4, IL-5 and IL-13 levels analysis (Bio-Techne, Minneapolis, CA, USA), according to the manufacturers’ protocol. The ELLA microfluidic platform automatically analysed the samples in triplicate.

Treatment safety was evaluated based on the incidence of adverse events (AEs) and laboratory safety parameters. AEs were assessed by clinical evaluation and were thoroughly evaluated for their relationship to study medication, intensity, and seriousness. Furthermore, laboratory safety parameters encompass safety blood measures, involving full blood count, at visit 1 and visit 2.

### 2.5. Statistical Analysis

Baseline variables were summarized as frequencies and percentages, means and standard deviations, or medians and ranges, as appropriate. T test, Wilcoxon-signed rank test or Fisher’s exact test were performed accordingly to determine statistical differences between the two treatment arms.

Accounting for any intercurrent events that occurred between patients’ follow-up visits, statistical analyses were performed on the modified per-protocol set (mPP), and modified intention-to-treat (mITT) analysis set. Intercurrent events were defined as any events during the treatment period that affected respiratory symptoms of patients due to external or confounding causes that are not related to the IMP, such as acute upper respiratory tract infections (URTI), and lack of compliance (<80%). In the mPP set, patients who encountered intercurrent events were treated as missing for analytical purposes [26]. Additionally, to reduce the variability due to temporal variations in plasma cytokines following cessation of IMP, patients with end of treatment and visit 2-time gap that exceeded 7 days were also removed. The mITT set on the other hand, contained all the patients, but patients who encountered intercurrent events had their outcome measures at visit 2 treated as missing for analytical purposes, and subsequently imputed using multilevel joint modelling multiple imputation method, in which 50 multiply imputes were generated. The analysis was carried out on all multiply datasets and consequently pooled using Rubin’s rules [27]. Additionally, across primary outcomes, mean change in scores (β) within treatment arms, across visit 1 and visit 2 was estimated using bootstrap paired T test in the mPP set, and paired T test in the mITT set.

To ascertain the treatment effects, generalized estimating equation (GEE) model was constructed for each primary outcome. The model included the “time” variable (comprising two timepoints: visit 1 and visit 2), along with the “arm” variable (comprising treatment allocation). The treatment effects were estimated by investigating the standardized arm-by-time interaction terms (β). The standardized coefficients, were determined by standardizing the primary outcome scores according to the mean and standard deviation, from all data points within the analysis set. An analysis of an adjusted model was also carried out by including baseline characteristics, which included baseline serum IgE levels. Baseline serum IgE levels were not standardized, and were only log-transformed to account for its right-skewed distribution. A full interaction GEE model was then constructed using the time, arm, and baseline IgE variables. Additionally, post hoc power testing was performed for the arm-by-time terms. This was done by extracting observed parameters extracted from the GEE models and linear mix effect models for multivariate normal data generation [28]. Simulations were conducted with 1000 iterations, and post hoc power were estimated as the proportion of replicates yielding a statistically significant arm-by-time interaction terms at α = 0.05 [29].

For the secondary endpoints analyses, paired bootstrapped T test was performed to ascertain changes in cytokine levels between visits. Additionally, association analysis using GEE was also performed to ascertain the relationship between cytokine level changes and symptom improvement. This was performed by constructing full interaction three-way interaction models, modelling patient reported outcome (TNSS) dependent on “time”, the “arm” variable, and the cytokine variable. Similar to the primary endpoint analysis, patient reported outcome scores were standardized according to the mean and standard deviation of the patient reported outcome score of the overall analysis set. Cytokine changes were not standardized, and its values were directly used for the construction of GEE models.

Safety analysis for AE occurrences was conducted on all patients, aside from those who were lost to follow up. Statistical test employed was Fisher’s exact test. Laboratory safety parameters were tested on the mPP set, using the crude GEE analysis described above.

For all analysis, level of significance was set to a two-tailed α = 0.05 and were performed using R statistical software (version 4.4.1, released on 14 June 2024).

## 3. Results

### 3.1. Baseline Characteristics

**Figure 1:**
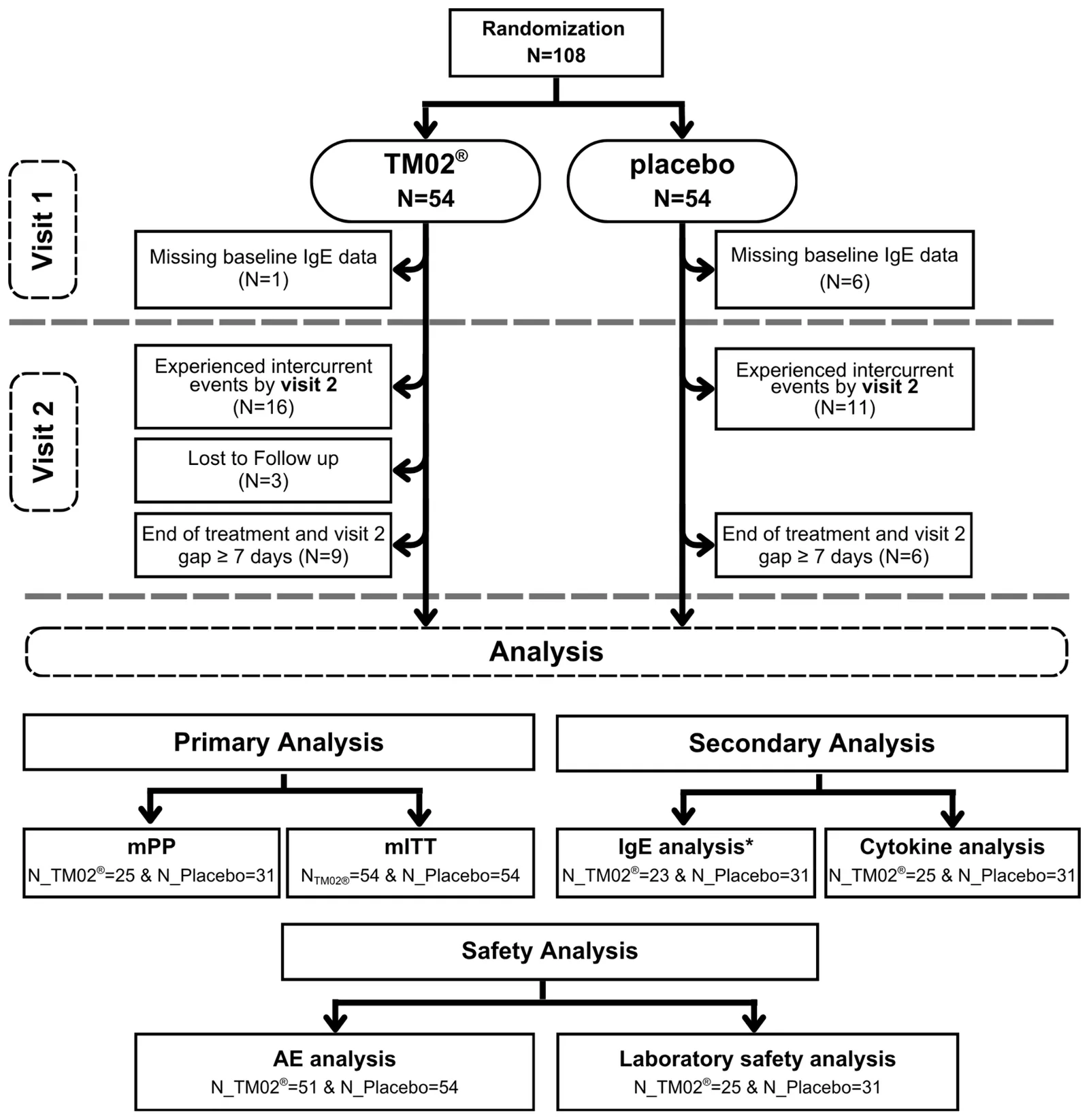
CONsolidated Standards of Reporting Trials (CONSORT) flow diagram of the study participants. * Two patients in the TM02^®^ arm had missing post-treatment IgE data.

A total number of 108 patients were screened and randomly allocated to treatment arms of equal size (N = 54 per arm). Baseline demographics, clinical characteristics, and baseline concomitant medications usage are displayed in Table 1 and demonstrated similar characteristics between the treatment arms with no statistical differences. At the end of the treatment period (visit 2; day 30), three patients were lost to follow up (N_TM02^®^ = 3; N_placebo = 0), another 27 patients experienced intercurrent events (N_TM02^®^ = 16; N_placebo = 11), and another 15 patients had end of treatment and visit 2 lag time exceeding 7 days (N_TM02^®^ = 9; N_placebo = 6). Of the patients who experienced intercurrent events, nine patients had <80% compliance (N_TM02^®^ = 6; N_placebo = 3), 15 experienced IP unrelated intercurrent events (N_TM02^®^ = 8; N_placebo = 7), and four patients had experienced other forms of intercurrent events (N_TM02^®^ = 2; N_placebo = 2). The summary of these protocol deviations is reported in Table S1. Additionally, seven patients had missing baseline serum IgE data, and was not included in the mPP set. This resulted in an arm size of 25 for the TM02^®^ arm and an arm size of 31 in the placebo arm, as demonstrated in Figure 1. It is important to note that of the analysis conducted using the mPP analysis set, the IgE analysis comprised two less patients owing to missing post-treatment serum IgE data, resulting in a total number of 54 (N_TM02^®^ = 23 and N_placebo = 31).

### 3.2. Efficacy

#### 3.2.1. Primary Endpoints with Patient Reported Outcomes

Analyses based on the mPP and mITT analysis sets yielded similar findings (Table S2). Therefore, only the results from the mPP are described here. The mPP analysis set comprised patients who did not have missing baseline IgE data, did not encounter intercurrent events, were not lost to follow up and had end of treatment to visit 2 gap of less than 7 days (N_TM02^®^ = 5; N_placebo = 31). As shown in Table 2, descriptive analysis showed statistically significant decrease in TNSS2wks and TNSS12hrs scores (total score, congestion, rhinorrhoea, sneezing and sleep) in the TM02^®^ arm. Additionally, the decrease in TNSS was estimated to be greater in the TM02^®^ arm (δ = −2.57) than in the placebo arm (δ = −1.93). A significant increase in ARCT total scores was observed in both treatment arms (Table 2). A similar trend of greater improvement was observed in the TM02^®^ arm, with TM02^®^ patients displaying a larger increase in ARCT scores (δ = 3.03; *p*-value < 0.001) compared to the placebo arm (δ = 2.03; *p*-value = 0.004).

**Figure 2:**
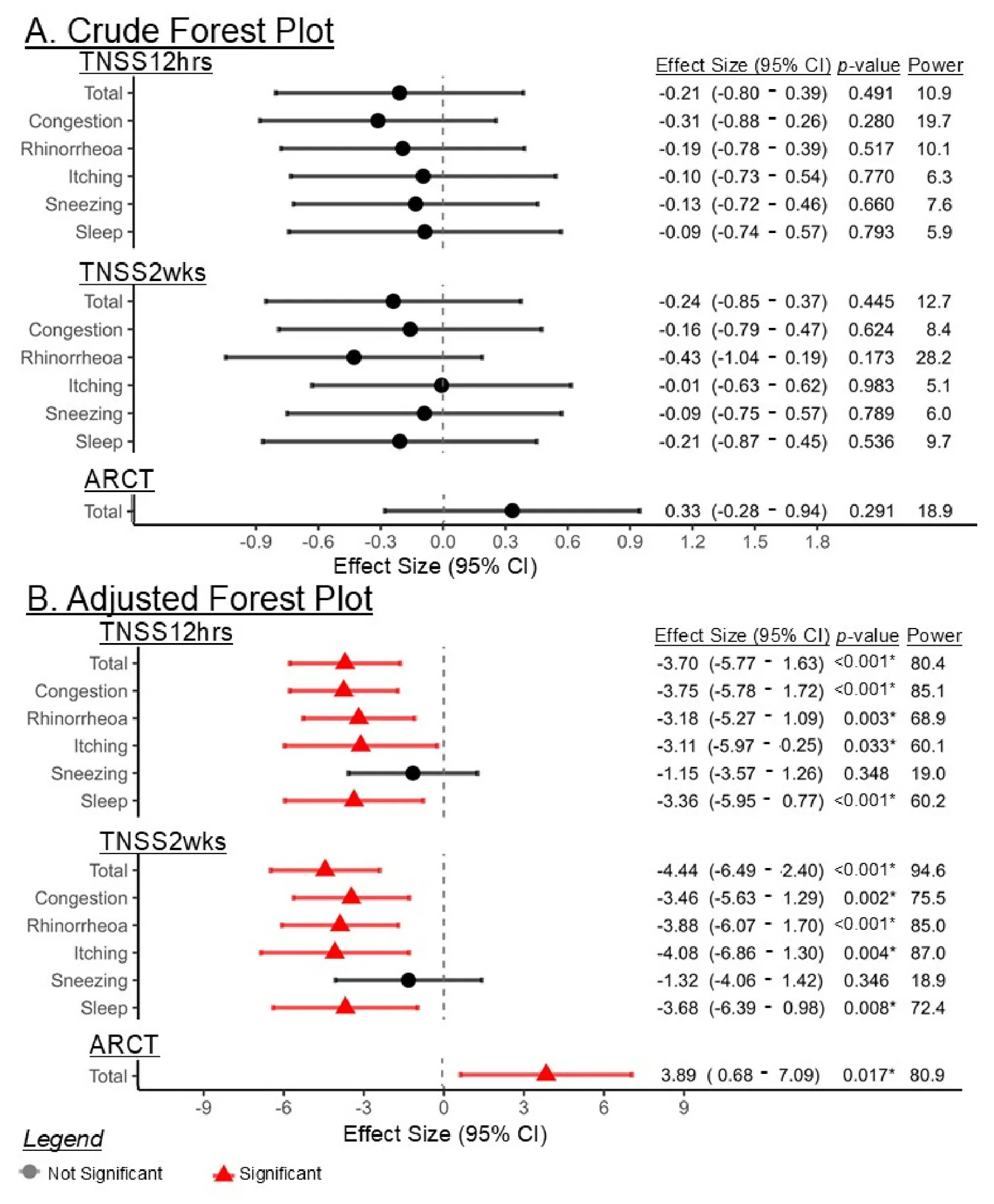
Forest plot of primary outcomes. The points represent the standardized coefficients of the arm-by-time interaction, and the error bars indicate 95% CI from the GEE models. Red triangular points depict coefficients that are significant (* *p*-value < 0.05), black circular points depict coefficients that are not significant.

Multivariate analysis with GEE was then employed to determine if the decrease in TNSS in the TM02^®^ arm was statistically stronger than the decrease in the placebo arm. The analysis revealed that none of the primary endpoints supported the hypothesis that TM02^®^ is superior to placebo in reducing TNSS (Figure 2 and Figure S1). Although the differences were not statistically significant, it can still be observed that the standardized effect sizes favor TM02^®^ arm, demonstrating patients in TM02^®^ arm are likely to have decreased TNSS over placebo arm. After adjusting for baseline serum IgE levels, significant treatment effects favouring TM02^®^ were observed for TNSS12hrs total score, TNSS2wks total score and ARCT score. Among TNSS12hrs scores, significant effects with *p*-value < 0.05 and post hoc power > 80.0% were nasal congestion symptom score reduction (β = −3.75; *p*-value < 0.001; post hoc power = 85.1%), followed by total symptom score reduction (β = −3.70; *p*-value < 0.001; post hoc power = 80.4%). Within TNSS2wks scores, statistical significances were present for total symptom score (β = −4.44; *p*-value < 0.001; post hoc power = 94.6%), rhinorrheoa (β = −3.88; *p*-value <0.001; post hoc power = 85.0%) and nasal itchiness (β = −4.08; *p*-value < 0.001; post hoc power = 87.0%). Lastly, the GEE analysis for ARCT score also revealed significant statistical improvement in the TM02^®^ over placebo (β = 3.89; *p*-value = 0.017; post hoc power = 80.9%).

#### 3.2.2. Secondary Analysis with Plasma Biomarkers

**Figure 3:**
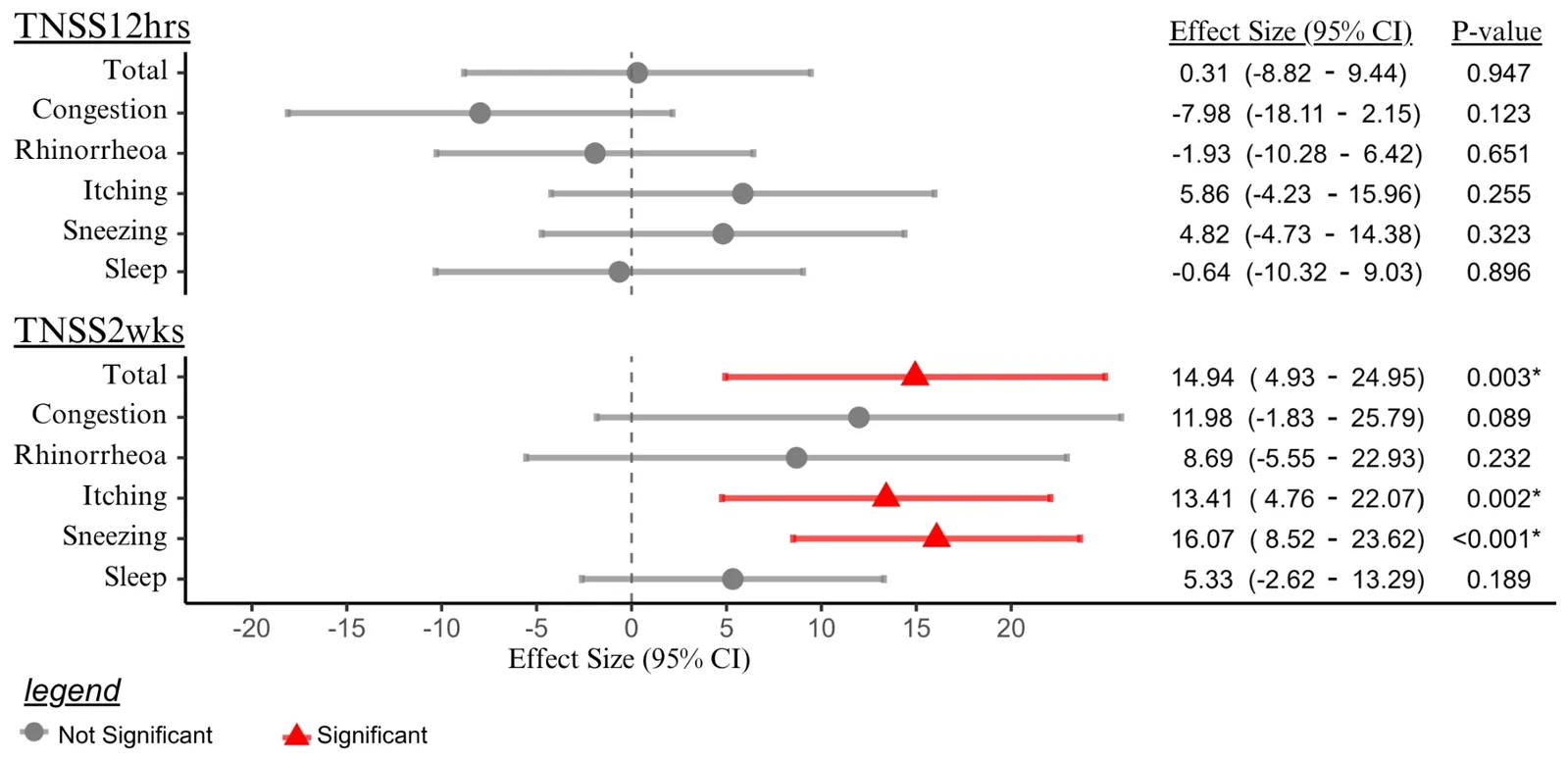
Secondary endpoints association analysis. The points represent the standardized coefficients of the arm-by-time-by-biomarker change interaction and the error bars indicate 95% CI from the GEE models. Red triangular points depict coefficients that are significant (* *p*-value < 0.05), black circular points depict coefficients that are not significant.

In the secondary analysis, serum IgE levels analysis was performed on the mPP set with an additional criterion to not include patients with missing post-treatment IgE data (patients with missing post-treatment IgE data were N_TM02^®^ = 2 and N_placebo = 0). This resulted in 54 patients (N_TM02^®^ = 23; N_placebo = 31). As shown in Table 3, there were no significant changes in the serum IgE levels in both arms. For plasma cytokine analysis, patients were from the mPP analysis set, comprising 56 patients (N_TM02^®^ = 25; N_placebo = 31). Low- and high-control cytokine mixtures were included in all runs and were within the manufacturer-established ranges, indicating consistency across batches. Cytokines that were detected below the lower limit of detection (LLOQ) or displayed high coefficient of variation (>30%) are tabulated in Table S3. In this analysis, we did not observe any significant changes in cytokine levels between treatment visits in either treatment arms, as shown in Table 3. However, a three-way interaction analysis using GEE demonstrated that IL-5 changes are positively associated with TNSS2wks scores in the TM02^®^ arm compared to the placebo arm, as shown in Figure 3. TNSS2wks’ total score, itching and sneezing scores displayed significant positive associations with IL-5 changes, with *p*-values of 0.003, 0.002 and <0.001, respectively. This implies that, for patients taking TM02^®^ who tend to display stronger symptom improvements for the past 2 weeks, they tend to also have stronger reductions in IL-5. Furthermore, this associative pattern is significantly associated with itching and sneezing symptoms.

### 3.3. Safety

Analysis of AEs was conducted on all patients, aside from patients who were lost to follow up (N_TM02^®^ = 3; N_placebo = 0). This resulted in 51 and 54 patients in the TM02^®^ arm and placebo arm, respectively. Total AEs, IMP-related AEs, and IMP-unrelated AEs were observed at similar frequencies between treatment arms without any statistical significance indicating difference in AE occurrences, as shown in Table 4. The details of adverse events are provided in Supplementary Table S4. Investigations into laboratory safety tests yielded a lack of statistical significance as well upon GEE analysis (Table S5).

## 4. Discussion

In the primary endpoints, we found that TM02^®^ significantly reduces nasal symptom severity in both the short term (TNSS12hrs total) and long term (TNSS2wks total). The placebo arm, however, also displayed reductions in nasal symptoms, albeit at weaker magnitudes of reduction. We also found patients’ capacity to manage AR (ARCT) to display similar trends as well, with significant improvements in both treatment arms, where TM02^®^ also displayed stronger magnitudes of improvement. This may be a result of a priori patient beliefs concerning the IMP or aspects relating to participation in a research study, or regression to the mean phenomenon [30,31]. Despite significant reductions in nasal symptoms and significant improvement of AR management in the placebo arm, the multivariate analysis revealed significant reduction in nasal symptoms and significant improvement in AR management in the TM02^®^ arm over placebo when accounting for baseline serum IgE levels. Serum IgE is strongly correlated and prognostic with nasal symptom severity in allergic rhinitis [32,33]. Therefore, accounting for this baseline, IgE allows the reduction in statistical variability and for a more generalizable interpretation of the present work [34,35]. The observed reductions in TNSS and improvements of ARCT suggest a clinically meaningful improvement in symptom burden and support the potential utility of TM02^®^ as an adjunctive therapy for patients with persistent AR.

In the context of molecular immunology, IFNγ levels are indicative of Th1 activation, while IL-4 and IL-13 levels are hallmarks of the Th2 activation [36,37,38]. Under normal physiologic conditions, Th1 and Th2 activations are in an equilibrium state, whereby increased Th1 activation leads to inhibition of Th2 and vice versa. In atopic diseases, including allergic rhinitis, the activation of Th2 responses due to exposure to environmental allergens leads to the skewing of Th2 responses and suppression of Th1 responses [5,36,37]. In persistent AR, this skewed Th2/Th1 state is prolonged and recalcitrant, resulting in constant and self-perpetuating allergic inflammation that ultimately leads to worse nasal symptoms [5,36]. In preclinical works pertaining to TM02^®^, it has been demonstrated that the fraction from TM02^®^ extract (xLr^®^) is capable of upregulating IL-6 secretion in RAW 264.7 cells. IL-6, in turn, is a key driver for T cell and B cell proliferation and differentiation [16]. Additionally, using the same cell line, the same study also demonstrated significant inhibition of IL-4 secretion [16]. In our present study, TM02^®^ demonstrated a trend towards modulating the IL-4, IL-13 and IFNγ levels, although these changes did not reach statistical significance. This suggests the potential ability of TM02^®^ to modulate immune cells, particularly by influencing Th1/Th2 cell differentiation.

A key driver bridging the molecular pathway between Th2 responses and allergic inflammation is IL-5 cytokine [36,39,40]. IL-5 is a pleiotropic cytokine secreted by various immune cells, including Th2 cells, that attracts, activates and prolongs eosinophils [36,40]. Higher levels of IL-5 and eosinophils directly contribute to worsened nasal symptoms, as a result of its granule secretions such as major basic protein (MBP) and eosinophil peroxidase (EPO) that lead to direct tissue damage [5,36]. Aside from the direct consequence of increased IL-5-driven eosinophilic inflammation, IL-5 has been demonstrated to modulate other areas of the immune system. Such cross-linking with other immune pathways has been revealed to include the promotion of IgE production in plasma cells, promotion of granulocyte survival, and even the suppression of regulatory T cells [41]. In our previous preclinical study using RAW 264.7 cells, TM02^®^ extract (xLr^®^) significantly altered IL-5 secretion [16]. Similarly, in another study utilizing a breast cancer xenograft model in nude mice, plasma IL-5 levels was altered following treatment with TM02^®^ extract (xLr^®^) and its fractions [17]. In the present study, we did not observe significant changes in IL-5 in the TM02^®^ arm. However, as demonstrated in the association analysis, significant associative patterns were identified between nasal symptom severity and IL-5 levels uniquely in the TM02^®^ arm. This is indicative that patients who responded more strongly towards TM02^®^ (larger magnitude of symptom reduction), were associated with greater reduction in IL-5 levels. Possible explanations for this discrepancy may include different biological systems, different sample collection protocols, different administration routes and complex immune system interplay. The present study is predicated upon real world human samples, which are subject to highly heterogenous and unpredictable genetic and environmental factors compared to highly controlled and predictable cell culture or animal models, resulting in the obfuscation of IL-5 changes [42]. Regarding sample collection protocols, the present study allowed time gaps of not more than 7 days between treatment ending and sample collection, which would lead to difficulty in finding the short-term changes in IL-5 which have a short biological half-life [43]. Additionally, the present study utilized oral route administration of the TM02^®^, which would subject the mushroom to various biotransformation processes in the gastroenteric tract (for example from the microbiota) and the first pass effect (from liver enzymes), that resulted in a different set of molecules that interacted with the immune cells [44,45,46,47]. Lastly, the immune system is known for its complex interplay and cross-talking between different immune units of cells, cytokines and immune pathways [5,48]. When administering TM02^®^ into a complete biological system with an intact immune system, as opposed to macrophage cell lines or T cell-lacking mice models, the net effect of TM02^®^ may have resulted in the suppression of IL-5 secretion, possibly due to the inhibition of T cell IL-5 secretion or paracrine signalling [48,49]. This difference may become more apparent in patients with persistent allergic rhinitis, who are constitutively exposed to allergens and consequently exhibit a Th2-skewed inflammatory immune state [6,50]. Administration of TM02^®^ in individuals with an established Th2/eosinophilic inflammatory state may have exerted a greater regulatory effect on this pathway, potentially resulting in an overall attenuation of IL-5-associated responses.

Regardless, the associative pattern highlights one potential mechanism of action underlying TM02^®^, where its nasal symptom-alleviating capabilities are dependent on IL-5-mediated mechanisms, which is related to eosinophils and allergic inflammation. While not all patients displayed significant reductions in allergic inflammation, this associative trend supports the possibility of allergic inflammation modulation as a potential mechanism of TM02^®^, corroborating the findings from the previous asthma clinical trial [18]. It is unclear as to why only strong responders to TM02^®^ are associated with IL-5 decline, while no associative patterns were identified in the measured Th1 and Th2 cytokines. Recent studies currently purport non-Th2-maintained allergic inflammation in allergic rhinitis and the potential prospects of therapies targeting solely the allergic-inflammatory pathways [41,51,52]. Cell surface markers of peripheral blood nuclear cells as a future endeavour would be able to shed light on this potential effect of TM02^®^ in modulating immune cells and allergic inflammation.

Furthermore, as a result of allergen exposure and prolonged Th2 activation, increased production of IgE, that subsequently binds to mucosal mast cells, leads to increased sensitization in immune cells for rapid allergic responses [32,33]. Overall, the higher levels of IgE led to worsened and prolonged AR symptoms. In the present study, we did not find serum IgE to significantly increase/decrease following treatment with TM02^®^.

Lastly, the safety of TM02^®^ was thoroughly investigated in the present study and did not reveal any major AEs or clinically concerning laboratory biomarkers (full blood count). Of the reported AEs, it is worth noting that the majority of TM02^®^-related AEs are mild, gastroenteric-related discomforts, such as bloatedness and constipation. Upon statistical analyses, we did not find TM02^®^ to significantly increase the likelihood of any AEs when compared against the placebo. Overall, the safety analysis in the present study is consistent with previous preclinical and clinical studies that demonstrated TM02^®^ to exhibit a favourable safety profile.

A limitation present in the study is concomitant medication usage. Patients recruited in the clinical trial are on at least one type of concomitant medication relevant to the treatment of AR, such as antihistamines or intranasal corticosteroids. Patients are mandated to be on at least one of these treatments due to ethical concerns where patients had to be on standard of care at the time of the study. Their concomitant medication use may have confounded the true extent of nasal symptom reduction and immune biomarker changes. While the present study does not demonstrate the use of TM02^®^ as a standalone alternative treatment option, the present study demonstrates its complementary usage in real world patients debilitated by the disease. Additionally, the analysis, especially mPP analysis, contained a lower number of patients, a discrepancy of 20 patients (N_TM02^®^ = 25, lower than the targeted 45). Consequently, the 44.4% attrition rate may have reduced statistical power and limited the precision of treatment effect estimates. The present study circumvented such potential attrition biases by implementing post hoc power testing to further screen results. While this approach is subjected to sampling variability and is not an equivalent to pre-determined power calculations, it improves the inferential capacity of the study by removing results that may appear significant entirely due to overestimation of the true effect [28,29].

To the best of our knowledge, the present study is the first randomised controlled trial to evaluate treatment effects of *Lignosus rhinoceros * TM02^®^, for the treatment of AR. Despite the aforementioned limitations, the present study was still able to determine a significant nasal symptom improvement effect of TM02^®^ without the occurrence of major AEs. Furthermore, we have uncovered a potential mechanism of action of the mushroom in the context of immune system modulation which builds upon both the novel discovery presented in this paper and our previously published review [53]. The findings from the present study support the potential use of TM02^®^ as a complementary or adjunctive treatment for AR.

## 5. Conclusions

This placebo-controlled, double-blinded clinical trial demonstrated that TM02^®^ significantly reduced the nasal symptoms of AR patients. This improvement was not accompanied by any major undesirable effects. Instead, TM02^®^ may have driven its nasal symptom reduction effect in a subset of patients via reduction in IL-5 and, consequently, allergic inflammation. Overall, TM02^®^ is a viable adjunctive or complementary treatment option for AR with persistent symptoms.

## Figures and Tables

**Table 1:** Demographics and clinical characteristics.

Characteristics	TM02^®^ (N = 54)	Placebo (N = 54)	*p*-Value
Age (years) (median (IQR))	31.60 (24.77–35.85)	30.85 (24.82–35.58)	0.890 ^‡^
Gender			
Female	30 (55.6%)	30 (55.6%)	1.000 ^§^
Male	24 (44.4%)	24 (44.4%)	1.000 ^§^
Ethnicity			0.488 ^∥^
Chinese	20 (37%)	21 (38.9%)	0.846 ^§^
Indian	6 (11.1%)	2 (3.7%)	0.270 ^§^
Malay	27 (50%)	29 (53.7%)	0.850 ^§^
Others	1 (1.9%)	2 (3.7%)	1.000 ^§^
BMI (median (IQR))	23.90 (21.75–27.85)	24.10 (22.05–28.62)	0.553 ^‡^
Rhinitis Assessment			
Mixed Rhinitis	27 (50%)	33 (61.1%)	0.342 ^§^
Allergic Rhinitis	27 (50%)	21 (38.9%)	0.342 ^§^
INCS Usage			0.504 ^∥^
None	21 (38.9%)	22 (40.7%)	1.000 ^§^
PRN	16 (29.6%)	11 (20.4%)	0.384 ^§^
Regular	17 (31.5%)	21 (38.9%)	0.554 ^§^
AH Usage			0.512 ^∥^
None	11 (20.4%)	7 (13.0%)	0.460 ^§^
PRN	39 (72.2%)	41 (75.9%)	0.831 ^§^
Regular	4 (7.4%)	6 (11.1%)	0.742 ^§^
Allergic Comorbidities			
Eczema	10 (18.5%)	14 (25.9%)	0.488 ^§^
Allergic Conjunctivitis	10 (18.5%)	11 (20.4%)	0.813 ^§^
Other Comorbidities	13 (24.1%)	13 (24.1%)	1.000 ^§^
Baseline IgE Levels (Log_10_ IU/mL) (median (IQR))	1.99 (1.68–2.48)	2.06 (1.79–2.44)	0.807 ^†^

^†^* p*-values determined using a T test; ^‡^* p*-values determined using Wilcoxon signed-rank test; ^§^* p*-values determined using Fisher’s exact test; ^∥^* p*-values determined using Chi-Square test. Statistical significance was defined as *p* < 0.05. Abbreviations: AH: antihistamine; BMI: body mass index; IQR: interquartile range; INCS: intranasal corticosteroids spray.

**Table 2:** Summary and descriptive analysis of primary endpoints.

	TM02^®^ (*n* = 25) ^‡^			Placebo (*n* = 31) ^‡^		
	Mean (SD)		−δ (95%CI) *p*-Value ^†^	Mean (SD)		−δ (95%CI) *p*-Value ^†^
	V1	V2	Change	V1	V2	Change
TNSS12hrs						
Total	4.52 (2.96)	1.96 (2.17)	2.57 (1.36–3.84) *p* < 0.001 *	5.00 (3.34)	3.06 (2.41)	1.93 (0.68–3.26) *p* = 0.002 *
Congestion	0.96 (0.79)	0.44 (0.65)	0.52 (0.20–0.84) *p* = 0.001 *	1.03 (0.75)	0.74 (0.63)	0.29 (0.03–0.55) *p* = 0.029 *
Rhinorrhoea	1.08 (0.70)	0.48 (0.59)	0.60 (0.28–0.92) *p* < 0.001 *	1.06 (0.93)	0.61 (0.62)	0.45 (0.13–0.77) *p* = 0.004 *
Itching	0.88 (0.93)	0.48 (0.59)	0.40 (0.00–0.80) *p* = 0.052	1.00 (0.89)	0.68 (0.75)	0.33 (−0.03–0.68) *p* = 0.084
Sneezing	1.08 (0.86)	0.36 (0.49)	0.72 (0.32–1.08) *p* = 0.002 *	1.29 (0.86)	0.68 (0.65)	0.61 (0.32–0.90) *p* < 0.001 *
Sleep	0.52 (0.71)	0.20 (0.41)	0.32 (0.08–0.60) *p* = 0.011 *	0.61 (0.84)	0.35 (0.71)	0.26 (−0.13–0.61) *p* = 0.226
TNSS2wks						
Total	6.80 (3.08)	4.36 (2.84)	2.45 (1.04–3.96) *p* = 0.006 *	6.87 (1.91)	5.10 (2.60)	1.77 (0.90–2.68) *p* < 0.001 *
Congestion	1.44 (0.82)	1.04 (0.68)	0.40 (0.04–0.76) *p* = 0.050 *	1.42 (0.62)	1.13 (0.62)	0.29 (0.06–0.52) *p* = 0.022 *
Rhinorrhoea	1.56 (0.71)	0.92 (0.76)	0.64 (0.28–1.00) *p* = 0.001 *	1.42 (0.67)	1.10 (0.70)	0.32 (0.03–0.58) *p* = 0.030 *
Itching	1.20 (0.62)	0.84 (0.69)	0.36 (−0.04–0.76) *p* = 0.099	1.45 (0.62)	1.10 (0.83)	0.35 (0.06–0.61) *p* = 0.037 *
Sneezing	1.64 (0.81)	1.12 (0.78)	0.52 (0.16 to 0.88) *p* = 0.016 *	1.58 (0.72)	1.13 (0.62)	0.45 (0.10–0.81) *p* = 0.015 *
Sleep	0.96 (0.84)	0.44 (0.58)	0.52 (0.12–0.92) *p* = 0.015 *	1.00 (0.77)	0.65 (0.84)	0.35 (−0.00–0.71) *p* = 0.072
ARCT Total Score	18.12 (2.54)	21.16 (2.37)	−3.03 (−4.40–−1.80) *p* < 0.001 *	17.58 (3.32)	19.61 (2.62)	−2.03 (−3.42–−0.71) *p* = 0.004 *

* Denotes statistical significance of *p*-value < 0.05. ^†^ Statistical significance determined using paired bootstrap T test; ^‡^ Patients who did not have missing baseline IgE data, did not encounter intercurrent events, were not lost to follow up and had end of treatment to visit 2 gap of less than 7 days. Abbreviations: ARCT: Allergic Rhinitis Control Test; CI: confidence interval; TNSS12hrs: Total Nasal Symptom Score 12hr; TNSS2wks: Total Nasal Symptom Score 2 weeks; V1: visit 1; V2: visit 2.

**Table 3:** Summary and descriptive analysis of secondary endpoints.

	TM02^®^ (N = 25) ^‡^			Placebo (N = 31) ^‡^		
	Median (IQR)		δ (95%CI) *p*-Value ^†^	Median (IQR)		δ (95%CI) *p*-Value ^†^
	V1	V2	Change	V1	V2	Change
Serum IgE (IU/mL) ^§^	78.00 (49.50–258.50)	79.00 (46.00–285.50)	0.81 (−21.92–20.87) *p* = 0.970	121.00 (67.00–268.50)	115.00 (63.50–258.50)	4.92 (−8.52–18.68) *p* = 0.510
IFNγ (pg/mL)	0.62 (0.48–0.92)	0.55 (0.42–0.85)	−0.23 (−0.68–0.10) *p* = 0.140	0.75 (0.51–1.16)	0.81 (0.61–1.13)	0.16 (−0.33–0.71) *p* = 0.514
IL-5 (pg/mL)	0.34 (0.21–0.47)	0.23 (0.17–0.43)	0.02 (−0.06–0.10) *p* = 0.642	0.41 (0.16–0.68)	0.28 (0.16–0.49)	1.77 (0.04–5.11) *p* = 0.157
IL-4 (pg/mL)	0.00 (0.00–0.00)	0.00 (0.00–0.00)	−0.14 (−0.38–−0.01) *p* = 0.075	0.00 (0.00–0.00)	0.00 (0.00–0.00)	0.00 (0.00–0.00) *p* = 0.153
IL-13 (pg/mL)	1.91 (0.28–5.68)	2.58 (0.00–5.40)	3.54 (−0.28–10.09) *p* = 0.108	2.07 (0.00–7.12)	2.45 (0.00–6.51)	−0.34 (−1.49–0.49) *p* = 0.425

Statistical significance (*p*-value < 0.05); ^†^ Statistical significance determined using paired bootstrap T test; ^‡^ Patients who did not have missing baseline IgE data, did not encounter intercurrent events, were not lost to follow up and had end of treatment to visit 2 gaps of less than 7 days (N_TM02^®^ = 25; N_placebo = 31); ^§^ Patients who did not have missing baseline or post-treatment IgE data, did not encounter intercurrent events, were not lost to follow up and had end of treatment to visit 2 gaps of less than 7 days (N_TM02^®^ = 23; N_placebo = 31). Abbreviations: IFNγ: interferon gamma; IgE: immunoglobulin E; IL: interleukin; IQR: interquartile range; V1: visit 1; V2: visit 2.

**Table 4:** Adverse events summary.

	TM02^®^ (N = 51) ^†^		Placebo (N = 54) ^†^		*p*-Value *
	Number of Events	Number of Patients N (%)	Number of Events	Number of Patients N (%)	
AEs	16	15 (29.4%) ^$^	17	16 (29.6%) ^$$^	1.000
IMP-related AEs	6	5 (9.8%) ^$^	5	5 (9.3%)	1.000
IMP-unrelated AEs	10	10 (19.6%)	12	11 (20.4%) ^$$^	0.815
Serious AEs	0	0 (0.0%)	0	0 (0.0%)	na
Deaths	0	0 (0.0%)	0	0 (0.0%)	na
Discontinued IMP due to an AE	0	0 (0.0%)	0	0 (0.0%)	na

^*^ Statistical analysis was performed on number of patients and statistical-significance-determined Fisher’s exact test. ^†^ Patients included in this analytical set are those who were not lost to follow up. ^$^ One patient reported slower bowel movement; one reported drowsiness; one reported bloatedness; one patient reported constipation; and one patient reported constipation and bloatedness. ^$$^ One patient reported constipation; one patient reported diarrhoea; one patient reported bloatedness; and two patients reported headaches. Abbreviations: AEs: adverse events; IMP: investigational medicinal product; na: not applicable.

## Data Availability

The data presented in this study are only available on request from the corresponding authors due to legal and ethical requirements, as stipulated in the participation information sheet and clinical trial agreement contract.

## Supplementary Materials

The following supporting information can be downloaded at: https://www.mdpi.com/article/10.3390/cimb48090898/s1.

## Abbreviations

The following abbreviations are used in this manuscript:

TNSS	Total Nasal Symptom Score
TNSS12hrs	TNSS reflective of past 12 h
TNSS2wks	TNSS reflective of past 2 weeks
ARCT	Allergic Rhinitis Control Test
IgE	Immunoglobulin E
IFN	Interferon
IL	Interleukin
AE	Adverse events
GEE	Generalized estimating equation
mPP	Modified per protoccol
USD	United States of America Dollars
Th2	T helper 2 cells
AH	Antihistamines
INCS	Intranasal corticosteroids
AIT	Allergen immunotherapy
MMRG	Medicinal Mushroom Research Group
MREC	Medical Research Ethics Council
IMP	Investigational medicinal product
ITS	Internal spacer region
NPVA	New Plant Variety Act
NPRA	National Pharmaceutical Regulatory Agency
EDTA	Ethylenediaminetetraacetic acid
UMMC	Universiti Malaya Medical Centre
mITT	Modified Intention-to-treat
URTI	Upper respiratory tract infection
IQR	Interquartile range
BMI	Body mass index
PRN	Pro re nata
IU	Internation units
V1	Visit 1
V2	Visit 2
CI	Confidence interval
SD	Standard deviation
