# Supplementary material for: Clinical Efficacy of TM02® in Allergic Rhinitis: Significant Symptom Alleviation Associated with IL-5 Downregulation and Reduced Allergic Inflammation
Source: Curr Issues Mol Biol. 2026 Sep 2;48(9):898. doi: 10.3390/cimb48090898 (PMC13605859; doi:10.3390/cimb48090898)
Supplement: Supplementary file 1 [file cimb-48-00898-s001.zip › cimb-4505537-supplementary.pdf]

current issues in molecular biology

MDPI

Supplementary Materials

Clinical Efficacy of TM02® in Allergic Rhinitis: Significant Symptom Alleviation Associated with IL-5 Downregulation and Reduced Allergic Inflammation

Hao-Dong Tan [1], Sien-Hui Tan [2], Boon-Hong Kong [1,*], Chee-Kuan Wong [3], Revadi Govindaraju [4], Tengku Ahmad Shahrizal Tengku Omar [2] and Shin-Yee Fung [1,*]

[1] Medicinal Mushroom Research Group (MMRG), Department of Molecular Medicine, Faculty of Medicine, Universiti Malaya, Kuala Lumpur 50603, Malaysia; haodong@um.edu.my
[2] Department of Otorhinolaryngology, Faculty of Medicine, Universiti Malaya, Kuala Lumpur 50603, Malaysia; shtan@ummc.edu.my (S.-H.T.); tshahrizal@ummc.edu.my (T.A.S.)
[3] Division of Respiratory Medicine, Department of Medicine, Faculty of Medicine, Universiti Malaya, Kuala Lumpur 50603, Malaysia; ckwong@um.edu.my
[4] Subang Jaya Medical Centre, Subang Jaya, Selangor 47500, Malaysia; grevadi@gmail.com
* Correspondence: kongbh@um.edu.my (B.-H.K.); syfung@um.edu.my (S.-Y.F.)

1. Protocol Adherence

Table S1. Summary of Protocol Deviations.

	TM02® (N=51) [†]	Placebo (N=54) [†]	P-value
IP Compliance (%) (median (IQR)) [‡]	100.00 (89.40 - 100.00)	100.00 (95.00 - 100.00)	0.145[§]
Adherent (≥80%)	44 (88.0%)	50 (94.3%)	0.515[‖]
Non-adherent (<80%)	6 (12.0%)	3 (5.7%)	0.515[‖]
Intercurrent events (non-IP related)	10 (19.6%)[$]	9 (16.7%)[$$]	1.000[‖]
Adverse events	8 (80.0%)	7 (77.8%)	1.000[‖]
Others	2 (20.0%)[$]	2 (22.2%)[$$]	1.000[‖]
End of treatment to visit 2 lag time (days) (median (IQR))	5.00 (4.00 - 6.00)	5.00 (3.25 - 6.00)	0.785[§]

[†] Patients included in this analysis group, are all randomized patients who were not lost to follow up; [‡] Two patients who are spouses inadvertently swapped their investigational product during the 30 days treatment period (IP mix up), leading to the inability to quantify their adherence. This resulted in the final sample size of 103 (N_TM02®=50 & N_Placebo=53); [§] P values determined Wilcoxon signed-rank test; [‖] P values determined Fisher's exact test; [$] One patient experienced IP mix-up and one patient subjected the IP to improper storage conditions, specifically placing it near the heat exhaust of an air conditioning compressor; [$$] One patient experienced IP mix-up and one patient experienced increased allergen exposure due to environmental changes associated with moving to a new house. Abbreviations: IP: Investigational product; IQR: interquartile range.

2. mITT analysis

Table S2. Primary Endpoints for mITT Analysis Group.   .

	TM02® (n=54) [‡]			Placebo (n=54) [‡]		
	Mean (SE)		-δ (95%CI) P-value[†]	Mean (SE)		-δ (95%CI) P-value[†]
	V1	V2	Change	V1	V2	Change
**TNSS12hrs**						
**5 Scale Total**	4.56 (0.88)	1.92 (0.72)	2.63 (1.64 - 3.62) P<0.001*	4.61 (0.80)	2.92 (0.72)	1.69 (0.73 - 2.64) P=0.001*
**4 Scale Total**	3.91 (0.73)	1.72 (0.70)	2.19 (1.30 - 3.08) P<0.001*	3.98 (0.67)	2.50 (0.63)	1.49 (0.69 - 2.28) P<0.001*
**Congestion**	1.04 (0.21)	0.47 (0.20)	0.57 (0.32 - 0.82) P<0.001*	1.00 (0.18)	0.74 (0.19)	0.26 (0.03 - 0.48) P=0.025*
**Rhinorrhoea**	0.94 (0.22)	0.45 (0.20)	0.49 (0.23 - 0.75) P<0.001*	0.93 (0.22)	0.54 (0.20)	0.39 (0.13 - 0.64) P=0.004*
**Itching**	0.87 (0.24)	0.51 (0.23)	0.36 (0.07 - 0.65) P=0.017*	0.98 (0.23)	0.64 (0.22)	0.34 (0.06 - 0.62) P=0.020*
**Sneezing**	1.06 (0.25)	0.40 (0.18)	0.65 (0.39 - 0.92) P<0.001*	1.07 (0.24)	0.61 (0.20)	0.46 (0.21 - 0.71) P=0.001*
**Sleep**	0.65 (0.24)	0.27 (0.17)	0.38 (0.13 - 0.63) P=0.003*	0.63 (0.22)	0.41 (0.20)	0.22 (-0.06 - 0.51) P=0.122
**TNSS2wks**						
**5 Scale Total**	6.72 (0.76)	4.21 (0.96)	2.51	7.20 (0.77)	5.29 (0.80)	1.92

			(1.43 - 3.59) P<0.001*			(1.02 -2.82) P<0.001*
**4 Scale Total**	5.72 (0.66)	3.73 (0.76)	1.99 (1.14 - 2.84) P<0.001*	6.06 (0.63)	4.55 (0.62)	1.50 (0.82 - 2.18) P<0.001*
**Congestion**	1.52 (0.21)	1.01 (0.26)	0.51 (0.21 - 0.82) P=0.002*	1.52 (0.19)	1.21 (0.20)	0.31 (0.07 - 0.55) P=0.013*
**Rhinorrhoea**	1.44 (0.20)	0.86 (0.25)	0.59 (0.30 - 0.87) P<0.001*	1.48 (0.21)	1.03 (0.21)	0.45 (0.19 - 0.71) P=0.001*
**Itching**	1.24 (0.21)	0.85 (0.25)	0.39 (0.10 - 0.68) P=0.009*	1.48 (0.21)	1.15 (0.24)	0.33 (0.08 - 0.58) P=0.011*
**Sneezing**	1.52 (0.20)	1.04 (0.27)	0.48 (0.20 - 0.76) P=0.001*	1.57 (0.22)	1.12 (0.21)	0.45 (0.16 - 0.75) P=0.003*
**Sleep**	1.00 (0.23)	0.51 (0.23)	0.49 (0.19 - 0.79) P=0.002*	1.15 (0.25)	0.77 (0.26)	0.38 (0.06 - 0.70) P=0.020*
**ARCT Total Score**	17.87 (0.89)	21.34 (0.89)	-3.47 (-4.55 - 2.40) P<0.001*	16.81 (0.91)	19.48 (0.93)	-2.67 (-3.84 - 1.50) P<0.001*

* Denotes statistical significance of P value < 0.05. † Statistical significance determined using paired bootstrap T test. ‡ Patients' visit 2 data were imputed if they had encountered intercurrent events or were lost - follow up. Abbreviations: ARCT: Allergic Rhinitis Control Test; CI: confidence interval; SE: Standard error; TNSS12hrs: Total Nasal Symptom Score 12hr; TNSS2wks: Total Nasal Symptom Score 2 weeks; V1: Visit 1; V2: Visit 2.

3. Immune Biomarker – ELLA Analysis

Table S3. Range and Variation of ELLA Cytokines.

Analyte	Detection Range	Visit 1		Visit 2	
		TM02® (N=25)	Placebo (N=31)	TM02® (N=25)	Placebo (N=31)
		Range of Detection			
IL-4	LLOQ	25 (100.0%)	31 (100.0%)	24 (96.0%)	31 (100.0%)
	N	0 (0.0%)	0 (0.0%)	1 (4.0%)	0 (0.0%)
IL-5	LLOQ	9 (36.0%)	12 (38.7%)	13 (52.0%)	15 (48.4%)
	N	16 (64.0%)	19 (61.3%)	12 (48.0%)	16 (51.6%)
IL-13	LLOQ	16 (64.0%)	20 (64.5%)	18 (72.0%)	23 (74.2%)
	N	9 (36.0%)	11 (35.5%)	7 (28.0%)	8 (25.8%)
IFNg	LLOQ	3 (12.0%)	2 (6.5%)	3 (12.0%)	3 (9.7%)
	N	22 (88.0%)	29 (93.5%)	22 (88.0%)	28 (90.3%)
		Coefficient of Variation			
IL-4	N	22 (88.0%)	31 (100.0%)	25 (100.0%)	30 (96.8%)
	!CV	3 (12.0%)	0 (0.0%)	0 (0.0%)	1 (3.2%)
IL-5	N	25 (100.0%)	31 (100.0%)	25 (100.0%)	31 (100.0%)
IL-13	N	21 (84.0%)	28 (90.3%)	23 (92.0%)	28 (90.3%)
	!CV	4 (16.0%)	3 (9.7%)	2 (8.0%)	3 (9.7%)
IFNg	N	25 (100.0%)	31 (100.0%)	25 (100.0%)	31 (100.0%)

Abbreviations: !CV: High coefficient of variation at >30%; LLOQ: Lower limit of detection; N: Within normal range.

4. Intercurrent Events

Table S4. Reported Adverse Events.

	TM02® (N=51) †		Placebo (N=54) †		P-value*
	Number of events	Number of patients N (%)	Number of events	Number of patients N (%)	
URTI	4	4 (7.8%)	6	6 (11.1%)	0.742
Constipation	2	2 (3.9%)	1	1 (1.9%)	1.000
Bloatedness	2	2 (3.9%)	1	1 (1.9%)	1.000
Migraine	1	1 (2.0%)	1	1 (1.9%)	1.000
Slower Bowel Movement	1	1 (2.0%)	0	0 (0.0%)	0.496
Sore Throat of Unknown Cause	1	1 (2.0%)	0	0 (0.0%)	0.496
Tooth Gumping	1	1 (2.0%)	0	0 (0.0%)	0.496
Anaphylactic Reaction	1	1 (2.0%)	0	0 (0.0%)	0.496
Drowsiness	1	1 (2.0%)	0	0 (0.0%)	0.496
COVID19 Infection	1	1 (2.0%)	0	0 (0.0%)	0.496
Food Poisoning	1	1 (2.0%)	0	0 (0.0%)	0.496
Headache	0	0 (0.0%)	2	2 (3.7%)	0.495
Hypotensive Episodes	0	0 (0.0%)	2	1 (1.9%)	0.496
Worsened Nasal Symptoms	0	0 (0.0%)	1	1 (1.9%)	0.496
Otitis Externa	0	0 (0.0%)	1	1 (1.9%)	0.496
Diarrhea	0	0 (0.0%)	1	1 (1.9%)	0.496
Paresthesia Episodes	0	0 (0.0%)	1	1 (1.9%)	0.496

* Statistical analysis was performed on number of cases and statistical significance determined Fisher's exact test. † Patients included in this analytical set, are those who were not lost to follow up. Abbreviations: URTI: Upper Respiratory Tract Infection.

5. Blood Safety analysis

Table S5. Safety Blood Safety Markers.

	TM02® (N=25) †			Placebo (N=31) †			Comparison with placebo
	Median (IQR)		δ (95%CI) P-value‡	Median (IQR)		δ (95%CI) P-value‡	β P value§
	V1	V2	Change	V1	V2	Change	
Hemoglobin (g/dL)	131.00 (124.00 - 147.00)	130.00 (124.00 - 144.00)	-0.06 (-3.64 - 2.92) P=0.866	137.00 (124.00 - 147.50)	136.00 (121.00 - 147.50)	2.01 (-0.16 - 4.19) P=0.083	2.014 P=0.324
Hematocrit (L/L)	0.39 (0.38 - 0.44)	0.40 (0.37 - 0.43)	-0.00 (-0.02 - 0.01) P=0.551	0.41 (0.38 - 0.44)	0.41 (0.36 - 0.44)	0.00 (0.00 - 0.01) P=0.226	0.007 P=0.307
MCV (fl)	84.00 (80.00 - 87.00)	83.00 (80.00 - 88.00)	-0.40 (-1.20 - 0.32) P=0.296	86.00 (81.50 - 88.00)	86.00 (82.00 - 88.00)	0.76 (-0.23 - 2.06) P=0.095	1.108 P=0.144
MCH (pg)	28.10 (26.20 - 28.70)	27.80 (26.10 - 28.70)	0.04 (-0.17 - 0.25) P=0.687	28.20 (26.60 - 29.75)	28.60 (26.65 - 29.55)	0.30 (-0.07 - 0.85) P=0.107	0.250 P=0.356
MCHC (g/L)	331.00 (324.00 - 338.00)	331.00 (321.00 - 337.00)	1.78 (-0.80 - 4.40) P=0.192	331.00 (322.50 - 339.50)	333.00 (321.00 - 338.50)	0.80 (-2.23 - 3.87) P=0.608	-1.049 P=0.605
White Blood Cell (10^9/L)	7.10 (5.90 - 8.30)	7.00 (6.40 - 8.20)	-0.10 (-0.63 - 0.37) P=0.679	7.40 (6.35 - 8.90)	7.20 (5.90 - 9.00)	0.27 (-0.22 - 0.80) P=0.269	0.111 P=0.714
Neutrophil (10^9/L)	3.83 (3.10 - 4.74)	3.87 (3.27 - 4.55)	-0.05 (-0.56 - 0.43) P=0.862	4.14 (3.33 - 5.98)	4.07 (3.31 - 5.68)	0.16 (-0.32 - 0.64) P=0.515	-0.083 P=0.770
Lymphocyte (10^9/L)	2.35 (1.88 - 2.52)	2.17 (1.93 - 2.51)	0.02 (-0.12 - 0.17) P=0.759	2.42 (2.01 - 2.86)	2.39 (1.81 - 2.69)	0.11 (-0.02 - 0.25) P=0.098	0.074 P=0.455
Eosinophil (10^9/L)	0.24 (0.12 - 0.45)	0.27 (0.16 - 0.51)	-0.07 (-0.20 - 0.03) P=0.169	0.21 (0.13 - 0.36)	0.21 (0.12 - 0.31)	0.02 (-0.02 - 0.06) P=0.293	0.090 P=0.118
Platelet (10^9/L)	310.00 (284.00 - 356.00)	302.00 (258.00 - 344.00)	-0.53 (-15.68 - 14.12) P=0.916	291.00 (263.00 - 373.50)	321.00 (242.00 - 358.50)	-0.64 (-14.35 - 12.84) P=0.932	-0.573 P=0.955

* Denotes statistical significance of P value < 0.05. † Patients who did not have missing baseline IgE data, did not encounter intercurrent events, were not lost to follow up and had end of treatment to visit 2 gap of less than 7 days. ‡ Statistical significance determined using paired bootstrap T test. § Statistical significance determined using crude Generalized Estimating Equation method. Abbreviations: MCH: Mean corpuscular hemoglobin; MCHC: Mean corpuscular hemoglobin concentration; MCV: Mean corpuscular volume; V1: Visit 1; V2: Visit 2.

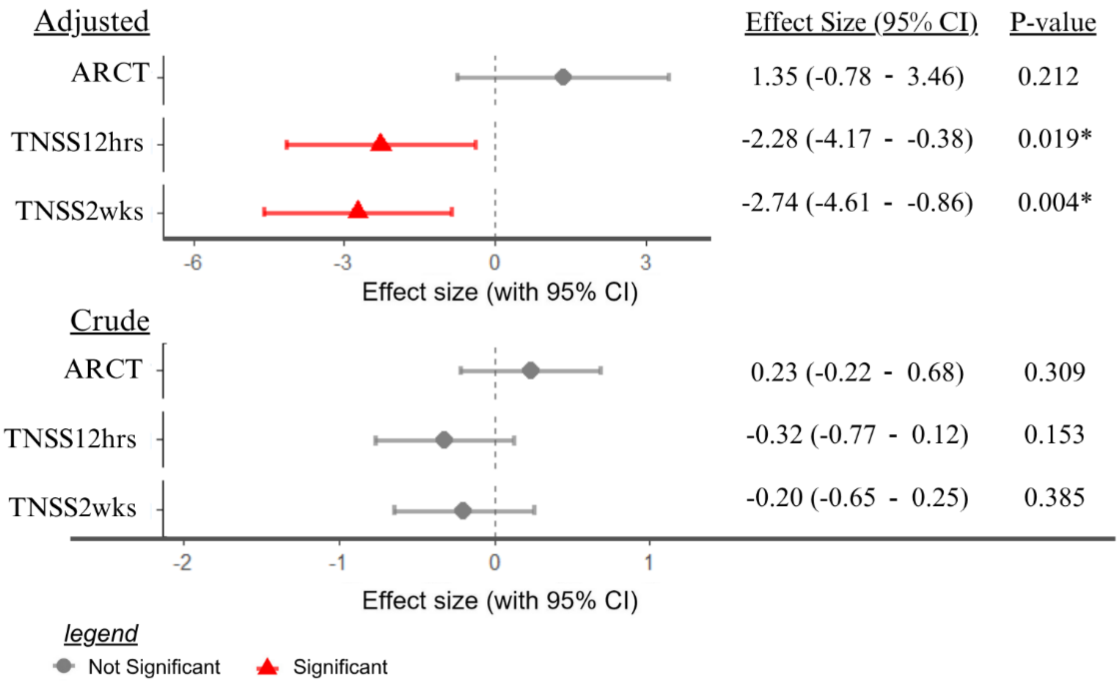

Figure S1. Forest plot of primary outcomes. The points represent the standardized coefficients of the arm-by-time interaction and the error bars indicate 95% CI from the GEE models. Red triangular points depict coefficients that are significant, black circular points depict coefficients that are not significant.
